# Teeth as an Indicator of the Environmental Exposure of Silesia Province’s Inhabitants in Poland to Metallic Trace Elements

**DOI:** 10.3390/toxics12010090

**Published:** 2024-01-20

**Authors:** Joanna Domagalska, Małgorzata Ćwieląg-Drabek, Grzegorz Dziubanek, Natalia Ulatowska, Sylwia Bortlik, Agata Piekut

**Affiliations:** 1 Department of Environmental Health, Faculty of Public Health in Bytom, Medical University of Silesia in Katowice, 18 Piekarska Street, 41-902 Bytom, Poland; mdrabek@sum.edu.pl (M.Ć.-D.); gdziubanek@sum.edu.pl (G.D.); apiekut@sum.edu.pl (A.P.); 2 Graduates of the Faculty of Public Health in Bytom, Medical University of Silesia in Katowice, 18 Piekarska Street, 41-902 Bytom, Poland; natula8925@gmail.com (N.U.); sylwia.bortlik@gmail.com (S.B.)

**Keywords:** environmental exposure, teeth, cadmium, lead, mercury, heavy metals, biomonitoring

## Abstract

(1) Background: The elemental composition of teeth can provide an estimate of environmental exposure to heavy metals. The aim of this study was to analyze the possibility of using teeth in the biomonitoring of environmental exposure to heavy metals as an indicator of contaminants present in the human residential environment. (2) Methods: The research materials were 110 samples of extracted teeth. The samples were taken from people living in three areas in the province of Silesia. The concentrations of cadmium, lead, and mercury in the samples were determined. (3) Results: The results of the chemical analysis of the collected samples showed a significant variation in the concentrations of heavy metals (Cd, Pb, and Hg) in the analyzed teeth. Furthermore, the mean concentrations of the analyzed heavy metals in the teeth varied according to the patient’s place of residence, the type of tooth analyzed, the presence of caries in the patient, and the smoking or non-smoking status of the patient. (4) Conclusions: The results of the chemical analysis of the teeth of inhabitants of three cities in the most polluted region of Poland indicate that they can be used as an indicator of environmental exposure to cadmium, lead, and mercury.

## 1. Introduction

In 2017, the World Health Organization (WHO) announced that exposure to toxic compounds in the human living environment, including heavy metals, is associated with up to 23% of deaths worldwide [1]. Lead (Pb), mercury (Hg), and cadmium (Cd) are ranked as the second, third, and seventh most hazardous substances, respectively, on the priority list compiled by the Heavy Metals Sub-Division of the Agency for Toxic Substances and Disease Registry and the European Environment Agency in 2019 [2,3,4]. According to The Lancet Commission on Pollution and Health, environmental pollutants, including particulate matter, persistent organic compounds, and heavy metals, contribute to more deaths than AIDS, tuberculosis, or malaria. In the European Union alone, this amounts to over 400,000 deaths annually. Globally, the number of deaths reached 9 million in 2017, accounting for 16% of all deaths. Air pollution, particularly in industrialized regions, is responsible for the deaths of approximately 280,000 Europeans annually. According to [5], the most polluted countries in Europe are Germany, Italy, the UK, France, and Poland. The province of Silesia in Poland is one of the most polluted regions due to its long history of heavy industry, including the mining and processing of non-ferrous metal ores in the southern part of the country [6,7,8]. In these regions, it is essential to continuously monitor the impact of environmental pollutants, such as heavy metals, on the health of the population.

Many studies suggest that various biological matrices, such as hair, fingernails, urine, blood, teeth, bones, or saliva [4,9,10,11,12,13,14,15,16,17,18,19], could be used as indicator materials for the continuous monitoring of environmental exposure to heavy metals. However, most of these matrices can only assess short-term (current) exposure levels. Urine and blood tests are most commonly used in clinical analyses. Determining the parameters of human exposure to heavy metals in these matrices is relatively simple and rapid. However, a major drawback of this method is the short persistence of metals, particularly lead, in such samples [20,21,22,23]. Pb is mainly deposited in bone, so the use of plasmas to monitor it appears to be inadequate and does not reflect the true level of exposure [22,24]. Cadmium is mainly deposited in the parenchymal organs (kidneys and liver), so blood Cd levels are also a poor reflection of whole-body Cd levels [24,25]. However, when it comes to hair and nails, heavy metals are not good biological indicators due to their susceptibility to the influence of external factors such as pollutants from dust deposited on the hair, hair care products, and styling products. The half-life of heavy metals in hair and nails ranges from a few months to several years depending on the metal analyzed [9,21,22,24,26]. Heavy metals have a significant affinity for bone and dental structures. These structures are chemically stable, and trace elements have the ability to replace calcium in the structure of hydroxyapatite, the building block of bones and teeth [9,11,27]. As toxic elements (especially heavy metals) accumulate in bone from the early stages of human skeletal development, bone tissue can be considered the most appropriate indicator for monitoring long-term exposures to pollutants in the human environment [9,11,12,24,28]. Teeth are a material very similar to bone tissue and are considered part of the human skeleton. The process of accumulation of toxic elements in dental tissues begins with the formation of tooth buds in the prenatal period. Both primary and permanent teeth (due to their similar structure) are reliable bioindicators of environmental exposure to heavy metals [9,11,12,24,27]. Furthermore, collecting tooth samples instead of bone offers a non-invasive approach to studying heavy metal levels in the human body. This method requires the use of milk teeth that have either fallen out naturally or that have been extracted for medical reasons.

Many studies on the relationship between the accumulation of metallic trace elements in human teeth and the presence of these pollutants in the environment have been published in recent years. However, the authors, fully aware of this fact, undertook this research. The area where the research was carried out (Silesia Province, Poland) is one of the most polluted areas in Europe, if not the world. Residents of this region have been exposed to heavy metals in above-standard concentrations for decades. The first study determining the concentration of selected heavy metals in the teeth of residents of Silesia Province was carried out in 2016. However, the study covered only two cities in the region, Bielsko-Biała and Ruda Śląska [11]. Another study, conducted by Moskalenko et al. [29], determined the concentration of lead in primary and permanent teeth of Silesian residents, indicating the need to expand the research to include additional metallic trace elements. The cited studies confirmed that teeth (in the phase of completed growth) can be a good source of information (bioindicator) regarding environmental exposure to elements such as cadmium, lead, or mercury. To confirm the results of the mentioned studies, Cd, Pb, and Hg were determined in the extracted teeth of residents of three selected cities in the Silesia Province, taking into account their concentrations based on age and gender, as well as the characteristics of the place of residence. The aim of this study was therefore to analyze the possibility of using teeth as a biomonitoring tool for environmental exposure to heavy metals. Tooth samples were collected from patients living in Silesia Province, one of the most polluted regions in Poland, in order to demonstrate the presence of pollutants in the human living environment.

## 2. Materials and Methods

### 2.1. Research Material

**Figure 1:**
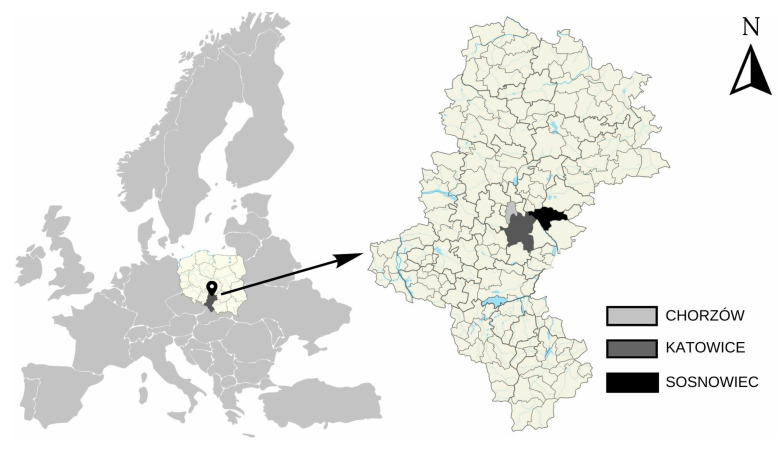
A section of a map of Poland showing the location of Chorzów, Katowice, and Sosnowiec.

The study used permanent human teeth from residents of three cities in Silesia Province that differ in location and air emissions: Katowice—the capital of Silesia Province, Chorzów—a city with one of the highest annual average concentrations of PM10 in the air in Poland, and Sosnowiec—a city located in the Dąbrowa Basin, in the eastern part of the Upper Silesian Industrial District. Figure 1 shows the precise geographical locations of the cities in Silesia Province. Silesia Province is one of the most economically and demographically developed regions in Poland. It is also described as the most urbanized area in Central and Eastern Europe, with the highest national average population density per 1 km^2^—355 people, which is significantly higher than the Polish average (121/km^2^) [30]. The Silesian agglomeration is one of the largest urbanized and industrialised areas in Central Europe, consisting of several cities and towns. The region has a long history of heavy industry, including mines, steelworks, and power plants. Currently, it is the last major coal mining area in the European Union. Historically, Katowice and Chorzów were part of Upper Silesia, and Sosnowiec is currently located in the province. However, the significant presence of heavy industry in the past distinguishes the city from others. Similar to other traditional industrial regions on the continent in the past, this region faces economic, environmental, and social challenges simultaneously.

The heavy metal content of 110 teeth from individuals aged 18–78 residing in Chorzów (30 samples), Katowice (30 samples), and Sosnowiec (50 samples) was tested. The samples were obtained from patients who had undergone tooth extraction for medical reasons, such as pain that could only be relieved by tooth extraction, advanced caries, or necessary procedures related to the start of orthodontic treatment. The teeth extracted included 23 incisors, 3 canines, 13 premolars, and 71 molars. This information, together with data on the patient’s sex, tooth type, and the presence of caries and plaque (this information was obtained from the patients’ medical records) on the extracted material, was included in the statistical analysis. Inclusion in the study was conditional on having no fillings or fillings other than amalgam. Additionally, all participants had lived in the specified city for at least 10 years.

As the study was not a medical experiment and involved the analysis of tooth samples that were waste (dental waste) from procedures performed for strictly medical reasons (unrelated to this study), bioethics committee approval was not required. Each study participant provided consent for the use of their extracted tooth for analysis.

### 2.2. Research Methods

#### 2.2.1. Sample Preparation for Analysis

The test to determine the heavy metal content of the teeth was carried out in the accredited analytical laboratory of the Department of Environmental Health of the Faculty of Public Health in Bytom of the Medical University of Silesia in Katowice.

The collected test material was carefully cleaned of soft tissue debris, rinsed with ultrapure water, and dried. The prepared tooth samples were placed in the grinding bowl of a LMW-S laboratory vibratory mill (TESTCHEM, Poland) and ground for 15 s. The ground sample was transferred to a Teflon container and weighed using a PS 750/X analytical balance (RADWAG, Radom, Poland).

#### 2.2.2. Sample Mineralization Process

To mineralize the samples in the Magnum II microwave mineralizer (ERTEC, Wrocław, Poland), 8 mL of concentrated spectrally pure 65% nitric acid (Merck, Rahway, NJ, USA) and 1 mL of 30% hydrogen peroxide were added to each sample.

The teeth were subjected to a two-stage pressure mineralization process. In the first stage, mineralization lasted 15 min (process parameters: 100% power, 43–45 bar pressure, and 295–300 °C temperature). In the second stage, the samples were cooled for approximately 10 min. At the end of the mineralization process, each sample was transferred to a 50 mL volumetric flask and ultrapure water was added to the specified volume (50 mL).

#### 2.2.3. Determination of Cadmium (Cd), Lead (Pb), and Mercury (Hg) Content

The concentration of heavy metals (Cd, Pb) in the samples was analyzed using a Savanta Sigma Atomic Absorption Spectrometer (ETAAS) with a PAL3000 automatic sample feeder (GBC, Keysborough, VIC, Australia) and a GF3000 graphite furnace (GBC, Australia). The concentration of mercury (Hg) in the teeth was analyzed using the cold vapor generation (CV) method in combination with atomic fluorescence spectrometry (AFS) in a total mercury analyzer (Millennium Merlin 10.025 (PS Analytical, Orpington, UK). Three parallel measurements were taken for each sample of Pb and Cd and two for mercury. The mean value was then calculated to determine the content of Pb, Cd, and Hg in mg/kg of tooth dry weight. The weight of the sample before mineralization, the volume of the test material, and its dilution were taken into account during the calculation.

### 2.3. Statistical Methods

Prior to conducting statistical analyses, the data on heavy metal concentrations in the teeth were categorized based on the study participants’ place of residence. The goodness-of-fit test and graphical interpretation of the results showed that the concentrations of all the metals analyzed did not have a normal distribution. Due to the different sample sizes provided by the residents of each city, a Kruskal–Wallis test was performed to identify significant differences between the mean metal concentrations in the samples. The non-parametric Mann–Whitney U test was used to determine the significance of differences between metal concentrations in samples by gender, taking into account the different numbers of samples provided by women and men. The significance of differences between metal concentrations in tooth samples provided by smokers and non-smokers and the presence or absence of caries was also tested. The Mann–Whitney U test was used for this purpose. The Kruskal–Wallis test was used to determine the significance of differences between metal concentrations in teeth according to their type: molars, premolars, incisors, or canines. The statistical analyses were conducted using Statistica 13.0 software at a 95% confidence level to indicate a significant result.

## 3. Results

The study included 110 participants aged 18 to 78 years. The mean age (±standard deviation) of the study participants from Katowice, Chorzów, and Sosnowiec was 36.3 ± 15.0 years (32.2 ± 12.6 years for females and 44.4 ± 16.7 years for males), 37.9 ± 15.6 years (38.4 ± 16.9 years for females and 36.8 ± 13.2 years for males), and 50.4 ± 13.8 years (53.5 ± 15.6 years for females and 45.7 ± 9.1 years for males), respectively. Considering the sex of the individuals from whom the tooth samples were obtained in each city, in Katowice, the percentage of women was 18.2% (20 samples) and men 9.1% (10 samples); in Chorzów, the percentage of women was 19.1% (21 samples) and men 8.2% (9 samples); and in Sosnowiec the percentage of women was 27.3% (30 samples) and men 18.1% (20 samples). 

Based on the age groups of the study participants, it can be concluded that the average concentration of the analyzed elements in the teeth varied. Statistically significant differences were demonstrated for cadmium and lead concentrations. Mercury concentrations did not differ significantly between the age groups (Table 1). The conducted post-hoc tests (Dunn’s test) allowed for statistically significant differences in the concentrations of individual elements to be determined between the age groups: for cadmium, for the 18–30 and 41–50 pair and the 18–30 and 51–60 pair (*p*-value lower than 0.001 and equal to 0.011, respectively), and in the case of lead, for the 18–30 and 41–50 pair, the 18–30 and 51–60 pair, the 18–30 and >60 pair, and the 31–40 and 41–50 pair (*p*-value lower than 0.001 and equal to 0.008, 0.017, and 0.004, respectively).

Table 2 shows the average concentrations of Cd, Pb, and Hg found in the teeth of individuals from the three study areas. The Kruskal–Wallis test results indicate that tooth samples from Sosnowiec residents had significantly higher concentrations of cadmium and lead compared to those from Katowice and Chorzów residents. Additionally, tooth samples from Chorzów residents had significantly higher concentrations of mercury than those from the other two cities.

The conducted post-hoc tests (Dunn’s test) allowed for statistically significant differences in the concentrations of individual elements to be determined between the cities: for mercury, such a difference was demonstrated for the Sosnowiec–Katowice and Katowice–Chorzów groups (*p*-value lower than 0.001 and equal to 0.008, respectively); for cadmium, this difference was demonstrated for the Sosnowiec–Chorzów, Sosnowiec–Katowice, and Katowice–Chorzów pairs (*p*-value lower than 0.001 for the first two pairs and 0.010 for the third pair); and in the case of lead, for the Sosnowiec–Chorzów and Sosnowiec–Katowice variables (*p*-value lower than 0.001 in both cases).

No statistically significant differences were found in the concentrations of the metals analyzed in tooth samples collected from males and females (Table 3). After considering the presence of dental caries in the study participants, the Mann–Whitney U test results showed no statistically significant differences between the concentrations of cadmium and lead in those with and without dental caries. However, significantly higher concentrations of mercury were found in those with dental caries (Table 4).

When analyzing teeth by type, the concentrations of cadmium and lead were significantly lower in molars compared to other types of teeth. Mercury concentrations did not differ significantly between the types of teeth studied (Table 5). The conducted post-hoc tests (Dunn’s test) allowed for statistically significant differences in the concentrations of individual elements to be determined between the teeth: for cadmium, it was determined for the molar–incisor and molar–premolar pairs (*p*-value equal to 0.001 in both cases), and in the case of lead, it was determined for the same pairs (*p*-value lower than 0.001 and equal to 0.011, respectively).

Survey participants were asked about cigarette smoking. The study revealed that cigarette smokers had significantly higher concentrations of cadmium and lead compared to non-smokers. However, no significant differences in mercury concentrations were found between the two groups (Table 6).

The study focused on cities located in an urban–industrial agglomeration. Due to variations in population size and industrial plant density across different areas of Upper Silesia, it is reasonable to assume that there would be variations in the concentrations of heavy metals found in the teeth samples and ambient air (Supplementary Material Table S1).

## 4. Discussion

The toxicity of heavy metals and their ability to accumulate in human organs and tissues makes it reasonable to introduce continuous monitoring to assess the risks to human health from environmental exposure to toxic elements. Biomonitoring is a scientifically accepted method for assessing health risks caused by environmental pollution in polluted areas. It involves analyzing the content of selected elements in biological materials such as hair, nails, urine, blood, and teeth. However, not all biological materials are suitable for assessing chronic environmental exposure to low levels of heavy metals [31,32]. Blood and urine, although widely used in such environmental studies, are not appropriate indicators of long-term exposure due to their short half-lives of 28–30 days [31,32]. Hair and nails have much longer half-lives, ranging from a few months to several years. However, it is important to note that cosmetic procedures such as hair dying or applying nail polish may interfere with the results of the analysis [24]. Teeth and bone appeared to be the best indicator materials, especially for long-term exposure, due to their very high affinity for heavy metals. However, teeth are a more accessible material than bone, as they are similar in composition and can be collected with minimal invasiveness during scheduled tooth extractions for medical reasons [24]. Analysis of the heavy metal content of tooth samples obtained from residents of polluted areas and from a population not exposed to environmental pollution confirmed that teeth are the best study material for determining the magnitude of the effects of environmental pollution on the human body [24,27,32].

It is important to consider that the study population was not specifically targeted. Therefore, factors such as the type of work performed, dietary habits, use of stimulants, dental status, and treatment, as well as the type of housing or fuel materials used, which can affect exposure to particulate matter and heavy metals, could potentially differentiate the results.

Analysis of the results obtained in this study showed that the concentrations of heavy metals (Cd, Pb, Hg) varied. The median concentrations of cadmium, lead, and mercury in the teeth of Katowice residents were 0.009 ± 0.013 mg Cd/kg, 6.708 ± 31.953 mg Pb/kg, and 5.543 ± 10.443 mg Hg/kg, respectively. The median concentrations of heavy metals in the teeth of the inhabitants of Chorzów were 0.047 ± 0.044 mg Cd/kg, 0.165 ± 0.397 mg Pb/kg, and 453.378 ± 1372.012 mg Hg/kg, respectively, whereas the median concentrations in the teeth of the inhabitants of Sosnowiec were 0.973 ± 1.940 mg Cd/kg, 27.147 ± 93.734 mg Pb/kg, and 0.375 ± 0.169 mg Hg/kg, respectively. In a study by Malara et al. the authors analyzed the content of heavy metals in molars and cortical bone samples taken at the time of wisdom tooth extraction in adult inhabitants of two towns in the Silesia Province (Bielsko-Biała and Ruda Śląska), similar to the towns selected in the present study [11]. The mean concentrations of Pb and Cd in the teeth of the inhabitants of Ruda Śląska (13.23 ± 1.93 mg Pb/kg and 0.12 ± 0.02 mg Cd/kg, respectively) and Bielsko-Biała (10.29 ± 1.90 mg Pb/kg and 0.10 ± 0.02 mg Cd/kg, respectively) were significantly higher than those of the inhabitants of Chorzów and Katowice. On the other hand, the median concentration of lead in the teeth of the inhabitants of Sosnowiec was two times higher than that of the teeth of the inhabitants of Ruda Śląska and 2.5 times higher than that of the teeth of the inhabitants of Bielsko-Biała. A similar relationship was found for cadmium concentrations in dental samples, where, in our study, the median concentrations of this element in the teeth of residents of Katowice and Chorzów were comparable to the concentrations found by Malara et al. in the teeth of residents of Ruda Slaska and Bielsko-Biala, whereas the median Cd concentrations in the teeth of residents of Sosnowiec were up to nine times higher [11]. The variation in heavy metal concentrations among residents of different cities may be attributed to differences in environmental exposure. Arruda-Neto et al. reached similar conclusions and noted that heavy metal content in teeth is influenced by lifetime exposure due to its ability to accumulate in the body [33]. Studies conducted in Poland and abroad have demonstrated that heavy metals such as Pb and Cd are present in higher concentrations in the teeth of patients residing in urban and polluted areas compared to those living in agricultural areas [11,27,33,34]. Similar conclusions were also presented by Kamberi et al. [35]. They determined the concentration of Pb in 86 permanent human teeth extracted from residents of three different geographical regions: Mitrovica and Klina in Kosovo and Graz in Austria. The teeth extracted from residents of Mitrovica had the highest lead levels, followed by those from Klina and Graz. The researchers emphasized that this difference in environmental pollution in the analyzed regions may be the cause [35].

Our study found no significant differences in heavy metal concentrations in the teeth of men or women in all cities analyzed. These results are consistent with those found by Malara et al. [11], who suggested that the lack of differences could be due to the small number of tooth samples analyzed. However, Asaduzzaman et al. reported that heavy metal concentrations in women’s teeth were generally higher than those in men’s teeth [27]. The concentration of Pb was significantly higher in the teeth of men than of women in studies by Fernández-Escudero et al. [36] and Al-Qattan and Elfawa [37].

Exposure to certain toxic metals may increase the risk of developing tooth decay. The study found significant differences in mercury concentrations in tooth samples from residents of all three cities who were diagnosed with tooth decay. In contrast, studies of heavy metal levels in Egyptian teeth showed a higher prevalence of dental caries in workers chronically exposed to lead. Indicators of dental caries were positively correlated with blood levels of this element in the study population [38].

The analysis revealed that the concentration of cadmium and lead in teeth may be influenced by the type of tooth. Statistically significant lower concentrations of cadmium and lead were found in molars compared to other tooth types. No similar relationship was found for mercury. In the study by Asaduzzaman et al. higher concentrations of Hg, Cu, and Sn were found in molars, whereas higher concentrations of Pb, Sr, Sb, and Zn were found in incisors. In addition, the study showed that the heavy metal content of teeth was statistically significantly dependent on whether the exposed person smoked cigarettes [27]. Heavy metal concentrations were higher in smokers, as confirmed by our own study in all three cities analyzed. The results of our own study confirm the above findings. The concentrations of cadmium and mercury in the teeth of cigarette smokers in all three cities analyzed were significantly higher than the concentrations of these metals in the teeth of non-smokers. Limited literature is available on the Hg content in the permanent teeth of the population. The authors suggest that occupational exposure is the primary risk factor for this metal. Certain occupations, such as those involving manufacturing, mining, or metalworking, may lead to increased exposure to heavy metals, which can result in accumulation in the body, including the teeth.

Assessing the impact of heavy metal contamination on human health can be challenging. One feasible method for evaluating environmental exposure to these compounds is through the use of biological indicators. The appropriate selection of biomarkers in environmental studies to assess the level of long-term exposure to heavy metals is important for primary prevention. Considering the reported levels of heavy metal concentrations in teeth, which reflect pollution from industrial emissions and urbanization, it is reasonable to consider human teeth a material that is a reliable bioindicator of environmental exposure. Analysis of heavy metal content in human teeth can provide information on long-term environmental exposure to these compounds. Tooth samples are easy to obtain during necessary medical procedures, and their storage does not require special conditions. Additionally, analysis of them is not complicated. However, planning a permanent monitoring system based on analyzing heavy metal content in teeth requires several issues to be addressed. These include determining the specific tooth type to analyze, collection method, tooth sample preparation, and analysis method. Each of these factors can have a significant impact on the obtained results. Establishing answers to these questions should form the basis for further analysis.

The absorption of heavy metals by teeth in individuals who lived in unpolluted areas until a less susceptible age may not be significant. It is important to note that residents of the Silesian agglomeration rarely relocate, and the participants in this study have lived almost exclusively in contaminated areas for most of their lives. There is a clear trend towards depopulation in Silesia rather than population growth. However, the findings of this study have to be seen in light of some limitations. Specifically, certain factors that could have influenced the results, such as occupational exposure, non-preferred dietary behavior, and exposure to secondhand smoke (including during childhood), were not considered. To ensure the accuracy of our findings, future research projects should verify them and exclude the possibility of additional exposure to heavy metals, such as through diet or occupation.

## 5. Conclusions

1. The chemical analysis of the teeth of inhabitants from three towns in the most polluted region of Poland (Silesia Province) suggests that they can serve as an indicator of environmental exposure to cadmium, lead, and mercury.

2. The results of the chemical analysis of the collected samples showed significant variations in the concentrations of heavy metals (Cd, Pb, and Hg) in the studied teeth. The average concentrations of the analyzed heavy metals in the teeth varied based on the patient’s place of residence, the type of tooth analyzed, the presence of caries in the patient, and whether the patient was a smoker or non-smoker. These correlations were statistically significant. 

3. Properly targeting preventive measures to reduce the health effects of heavy metal exposure in residents of polluted areas requires continuous biomonitoring of the levels of these compounds in the body. Non-invasive sampling methods should be used for testing.

## Figures and Tables

**Table 1:** Heavy metal concentrations (mg/kg) in patients’ teeth by age group.

Age Group	*n*	Heavy Metal Concentrations in Teeth (mg/kg)								
		Cd			Pb			Hg		
		Me	Min–Max	*p*-Value *	Me	Min–Max	*p*-Value *	Me	Min–Max	*p*-Value *
18–30	33	0.021	0.0004–1.339	<0.001 ^a^	0.093	0.0035–14.270	<0.001 ^a^	0.470	0.0239–80.706	0.874
31–40	19	0.045	0.0004–1.126		0.574	0.0035–13.430		0.417	0.0094–4500.0	
41–50	25	0.339	0.0004–9.086		9.110	0.0035–558.42		0.500	0.0407–5.039	
51–60	17	0.457	0.0004–1.133		6.360	0.0035–22.390		0.500	0.0568–4500.0	
>60	16	0.126	0.0004–2.259		4.430	0.0035–14.720		0.411	0.028–4500.0	

*n*—number of samples, Me—median, Min—minimum, Max—maximum. *—*p*-values, estimated using the Kruskal–Wallis test and used to identify statistically significant differences between the mean heavy metal concentrations in the tooth samples of patients in the different age groups. ^a^—indicates that the heavy metal concentrations in the samples from patients in the different age groups were significantly different.

**Table 2:** Descriptive statistics for the heavy metal concentrations (mg/kg) in teeth.

Heavy Metal	Samples from People Living in Katowice (n = 30)		Samples from People Living in Chorzów (n = 30)		Samples from People Living in Sosnowiec (n = 50)		*p*-Value *
	Me	Min–Max	Me	Min–Max	Me	Min–Max	
Cd	0.009	0.0004–0.053	0.047	0.0004–0.224	0.973	0.057–11.126	<0.001 ^a^
Pb	6.708	0.004–175.525	0.165	0.004–1.616	27.147	0.400–558.420	<0.001 ^a^
Hg	5.543	0.041–48.141	453.378	0.009–4500.0	0.75	0.028–0.500	<0.001 ^a^

n—number of samples, Me—median, Min—minimum, Max—maximum. *—*p*-values, estimated using the Kruskal–Wallis test and used to identify statistically significant differences between the mean heavy metal concentrations in the samples provided by people living in Katowice, Chorzów, and Sosnowiec. ^a^—indicates that the heavy metal concentrations in the samples provided by people living in Katowice, Chorzów, and Sosnowiec were significantly different.

**Table 3:** Heavy metal concentrations (mg/kg) in the samples provided by women and men.

Heavy Metal	Teeth (n = 110)			
	Women (n = 71) Median	Men (n = 39) Median	U-Value	*p*-Value *
Cd	0.055	0.174	1120.500	0.099
Pb	0.517	3.180	1152.000	0.147
Hg	0.500	0.408	1096.500	0.072

n—number of samples. *—*p*-values, estimated using the Mann–Whitney U test and used to identify statistically significant differences between the metal concentrations in the samples provided by women and men.

**Table 4:** Heavy metal concentrations (mg/kg) in tooth samples with and without caries.

Heavy Metal	Teeth (n = 110)			
	Dental Caries			
	Yes (n = 82) Median	No (n = 28) Median	U-Value	*p*-Value *
Cd	0.061	0.062	1075.500	0.621
Pb	1.232	1.019	999.000	0.308
Hg	0.500	0.159	727.500	0.004 ^a^

n—number of samples. *—*p*-values, estimated using the Mann–Whitney U test and used to identify statistically significant differences between the metal concentrations in the samples with and without dental caries. ^a^—indicates that the mean concentrations in the samples with caries and without dental caries were significantly different.

**Table 5:** Heavy metal concentrations (mg/kg) in tooth samples.

Heavy Metal	Teeth (n = 110)					
	Type of Tooth					
	Incisors (n = 23) Median	Canines (n = 3) Median	Premolar (n = 13) Median	Molars (n = 71) Median	H-Value	*p*-Value *
Cd	0.339	0.247	0.521	0.029	23.835	<0.001 ^a^
Pb	9.920	5.790	6.560	0.150	27.283	<0.001 ^a^
Hg	0.452	0.409	0.500	0.500	0.428	0.934

n—number of samples. *—*p*-values, estimated using the Kruskal–Wallis test and used to identify statistically significant differences between the metal concentrations in the different tooth types. ^a^—indicates that the mean concentrations in the samples of different types of teeth were significantly different.

**Table 6:** Heavy metal concentrations (mg/kg) in tooth samples of smokers and non-smokers.

Heavy Metal	Teeth (n = 110)			
	Smoking			
	Yes (n = 50) Median	No (n = 60) Median	U-Value	*p*-Value *
Cd	0.323	0.043	1126.500	0.025 ^a^
Pb	4.400	0.185	981.500	0.002 ^a^
Hg	0.500	0.409	1273.000	0.174

n—number of samples. *—*p*-values, estimated using the Mann–Whitney U test and used to identify statistically significant differences between the metal concentrations in the samples provided by smokers and non-smokers. ^a^—indicates that the mean concentrations in the samples provided by smokers and non-smokers were significantly different.

## Data Availability

The data that support the findings of this study are available from the corresponding author upon reasonable request.

## Supplementary Materials

The following supporting information can be downloaded at: https://www.mdpi.com/article/10.3390/toxics12010090/s1. Table S1. The median concentration of heavy metals in the teeth of residents of three cities, and PM10, Pb, and Cd in the air.
