# Supplementary material for: Teeth as an Indicator of the Environmental Exposure of Silesia Province’s Inhabitants in Poland to Metallic Trace Elements
Source: Toxics. 2024 Jan 20;12(1):90. doi: 10.3390/toxics12010090 (PMC10818862; doi:10.3390/toxics12010090)
Supplement: Supplementary file 1 [file toxics-12-00090-s001.zip › toxics-2762638-supplementary.pdf]

Supplementary Materials

Table S1. The median concentration of heavy metals in the teeth of residents of three cities, and PM_{10}, Pb, and Cd in the air.

City	Heavy metal concentrations in teeth [mg/kg]			Heavy metal concentrations in air (1989-2008)		
	Hg	Pb	Cd	PM_{10} [μg/m^3]*	Pb [ng/m^3]*	Cd [ng/m^3]*
**Chorzów**	453.378	0.165	0.047	98.260	274.344	7.055
**Katowice**	5.543	6.708	0.009	82.430	219.200	5.195
**Sosnowiec**	0.75	27.147	0.973	86.030	191.388	5.940

*source: data from 1989-2001 - Provincial Sanitary and Epidemiological Station in Katowice, Poland; data from 2002-2008 - Provincial Inspectorate of Environmental Protection in Katowice.
